# Assessment of the causal relationship between gut microbiota and cardiovascular diseases: a bidirectional Mendelian randomization analysis

**DOI:** 10.1186/s13040-024-00356-2

**Published:** 2024-02-26

**Authors:** Xiao-Ce Dai, Yi Yu, Si-Yu Zhou, Shuo Yu, Mei-Xiang Xiang, Hong Ma

**Affiliations:** 1 Department of Cardiology, The Second Affiliated Hospital, School of Medicine, Zhejiang University, State Key Laboratory of Transvascular Implantation Devices, Cardiovascular Key Laboratory of Zhejiang Province, Hangzhou, Zhejiang 310009 China; 2 Department of Anesthesiology, The Second Affiliated Hospital, School of Medicine, Zhejiang University, Hangzhou, 310009 China

**Keywords:** Cardiovascular diseases, Gut microbiota, Mendelian randomization analysis

## Abstract

**Background:**

Previous studies have shown an association between gut microbiota and cardiovascular diseases (CVDs). However, the underlying causal relationship remains unclear. This study aims to elucidate the causal relationship between gut microbiota and CVDs and to explore the pathogenic role of gut microbiota in CVDs.

**Methods:**

In this two-sample Mendelian randomization study, we used genetic instruments from publicly available genome-wide association studies, including single-nucleotide polymorphisms (SNPs) associated with gut microbiota (*n* = 14,306) and CVDs (*n* = 2,207,591). We employed multiple statistical analysis methods, including inverse variance weighting, MR Egger, weighted median, MR pleiotropic residuals and outliers, and the leave-one-out method, to estimate the causal relationship between gut microbiota and CVDs. Additionally, we conducted multiple analyses to assess horizontal pleiotropy and heterogeneity.

**Results:**

GWAS summary data were available from a pooled sample of 2,221,897 adult and adolescent participants. Our findings indicated that specific gut microbiota had either protective or detrimental effects on CVDs. Notably, Howardella (OR = 0.955, 95% CI: 0.913–0.999, *P* = .05), Intestinibacter (OR = 0.908, 95% CI:0.831–0.993, *P* = .03), Lachnospiraceae (NK4A136 group) (OR = 0.904, 95% CI:0.841–0.973, *P* = .007), Turicibacter (OR = 0.904, 95% CI: 0.838–0.976, *P* = .01), Holdemania (OR, 0.898; 95% CI: 0.810–0.995, *P* = .04) and Odoribacter (OR, 0.835; 95% CI: 0.710–0.993, *P* = .04) exhibited a protective causal effect on atrial fibrillation, while other microbiota had adverse causal effects. Similar effects were observed with respect to coronary artery disease, myocardial infarction, ischemic stroke, and hypertension. Furthermore, reversed Mendelian randomization analyses revealed that atrial fibrillation and ischemic stroke had causal effects on certain gut microbiotas.

**Conclusion:**

Our study underscored the importance of gut microbiota in the context of CVDs and lent support to the hypothesis that increasing the abundance of probiotics or decreasing the abundance of harmful bacterial populations may offer protection against specific CVDs. Nevertheless, further research is essential to translate these findings into clinical practice.

**Supplementary Information:**

The online version contains supplementary material available at 10.1186/s13040-024-00356-2.

## Introduction

Cardiovascular diseases (CVDs) are phenomena that affect the heart and blood vessels, including ischemic heart disease and stroke. In most countries, including China, CVD-related mortality and morbidity are increasing. CVD has become the leading cause of death and premature death in China, accounting for 40% of deaths in the Chinese population [1–3]. The risk factors for CVD include hypertension, type 2 diabetes, and unhealthy lifestyles. Evidence from an increasing number of studies [4, 5] on unfavorable lifestyles has revealed, that different dietary habits can lead to changes in the levels of gut microbiota and the concentrations of their metabolites. In recent studies, Deng et al. found that empagliflozin may provide cardiovascular benefits by altering gut microbiota and plasma metabolites [6]. Consistently, Telle-Hansen et al. showed that replacing saturated fatty acids with polyunsaturated fatty acids altered heart-related the abundance of specific gut microbiota [7], which improved the prognosis. Several other studies have shown that changes in trimethylamine N-oxide (TMAO) which is produced by gut bacteria, strongly associated with different pathological conditions in the heart, including hypertension and atherosclerosis [8, 9]. Hence, TMAO has been considered not only as a novel biomarker for CVD but also a therapeutic target for chronic disease [10]. These studies therefore demonstrate the strong relationship between gut microbiota and CVDs as well as the risk factors for CVDs.

The intestinal flora is mainly composed of bacteria; it also includes fungi and viruses. Intestinal flora participate in the formation and regulation of the intestinal mucosal barrier, control nutrient intake and metabolism, assist in the maturation of immune tissues, and prevent the reproduction of pathogenic microorganisms [11–15]. Numerous studies have reported interactions between gut microbiota and diseases, such as cerebral ischemia–reperfusion injury, hepatic fibrosis, and CVD [16–18]. Indeed, proper gut microbiota structure and metabolite function are critical for the maintenance of homeostasis. If the gut microbiota is out of balance, it can lead to atherosclerosis, high blood pressure, heart failure and cardiac arrhythmias [19]. Although many studies have reported these effects, all of the gut microbiota related to CVDs have not been identified. Because the instruments, equipment and experimental subjects vary across studies, there is no consensus in the conclusions drawn from CVDs research. Therefore, the causal relationship between gut microbiota and CVD requires further investigation.

This study aimed to establish a causal relationship between gut microbiota and cardiovascular disease outcomes through genome-wide association studies (GWAS) and Mendelian randomization (MR). In this study, MR was used to elucidate the causal effect of gut microbiota on cardiovascular disease and to examine whether the effect is bidirectional.

## Methods

### Two-sample bidirectional MR design

This study used a two-sample bidirectional MR design with single-nucleotide polymorphisms (SNPs) as instrumental variables (IVs) to assess the causal relationship between gut microbiota and cardiovascular disease. The IVs were required to meet three criteria: IVs should be strongly associated with the exposure; IVs should be associated with the exposure outcome without confounding; and IVs should only affect the outcome through the exposure [20].

### Ethical review

All published genome-wide association studies (GWAS) and received ethic approval from the appropriate institutional review board. Only abstract-level data were used in this study, and therefor, no additional ethics approval was needed.

### Exposure data

SNPs related to gut microbiota composition were selected as IVs from the IEU Open GWAS project (https://gwas.mrcieu.ac.uk/; updated to 2023.06.26). The study population was predominantly adults and adolescents of European ancestry, with a total sample size of 14,306 individuals across 24 cohorts. We obtained data on 121 gut microbial taxa at the genus level.

### Outcome data

We defined cardiovascular diseases as atrial fibrillation (AF), coronary artery disease (CAD), myocardial infarction (MI), ischemic stroke (cardioembolic; IS) and hypertension and then downloaded data that were reported in the IEU Open GWAS project (https://gwas.mrcieu.ac.uk/; updated to 2023.06.26). The GWAS examined the following CVDs: AF (*n* = 1,030,836), CAD (*n* = 547,261), MI (*n* = 395,795), IS (*n* = 211,763), and hypertension (*n* = 21,936).

### Instrumental variable selection

**Fig. 1:**
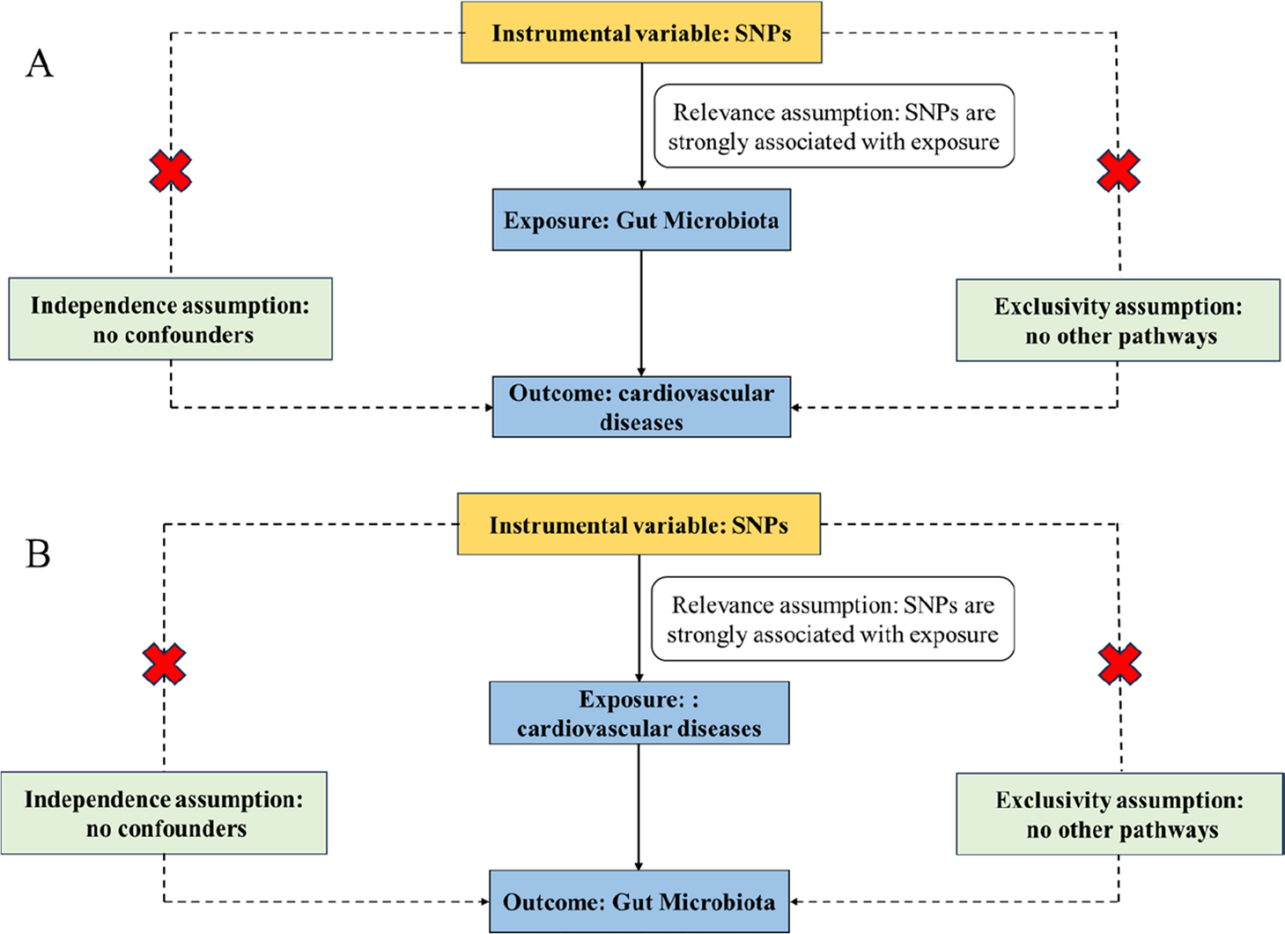
Flowchart of the two-sample MR-analysis process. **A** Three important hypotheses for gut microbiota of cardiovascular diseases by forward MR. These three paths represent these three different hypotheses. Relevance assumption: SNPs are associated with gut microbiota (the exposure). Independence assumption: SNPs were completely independent of any potential confounders affecting gut microbiota and cardiovascular diseases. Exclusivity assumption: SNPs affect metabolites only through gut microbiota (exposure) and not via any alternative causal pathways. **B** Three important hypotheses for cardiovascular diseases of gut microbiota by forward MR. These three paths represent these three different hypotheses. Relevance assumption: SNPs are associated with cardiovascular diseases (the exposure). Independence assumption: SNPs were completely independent of any potential confounders affecting gut microbiota and cardiovascular diseases. Exclusivity assumption: SNPs affect metabolites only through cardiovascular diseases (exposure) and not via any alternative causal pathways

The flowchart of this study is shown in Fig. 1. In short, cardiovascular disease is the outcome when the gut microbiota is the exposure factor and vice versa.

Bacterial taxa were analyzed at the genus level. To ensure the authenticity and accuracy of the conclusions on the causal link between the gut microbiota and cardiovascular diseases, the following quality control steps were used to select the optimal IVs. (1) If few significant SNPs were available, SNPs associated with a relaxed statistical threshold (*P* < 1.0 × 10^–6^) were selected. (2) The 1000 Genomes Project European sample data were used as a reference panel to calculate linkage disequilibrium (LD) among SNPs, and among those SNPs with* r*^2^ < 0.001 (aggregation window size = 10,000 kb), only the SNP with the lowest *P* value was retained. (3) SNPs with a minor allele frequency (MAF) ≤ 0.01 were removed. (4) When palindromic SNPs are present, forward-strand alleles were inferred using allele frequency information.

### Statistical analysis

In this study, multiple methods including inverse variance weighting (IVW), MR Egger regression, weighted median, simple model, MR-PRESSO, and weighted model were used to examine the causality between gut microbiota and cardiovascular disease. For each potential effect, we incorporated the MR estimates using inverse variance weighted (IVW) meta-analysis, which essentially translates into a weighted regression of the SNP outcome effect on the SNP exposure effect, with the intercept constrained to zero. The IVW method used a meta-analysis approach combined with the Wald estimates for each SNP to obtain an overall estimate of the effect of gut microbiota on cardiovascular diseases. Similarly, if an instrumental SNP shows horizontal pleiotropy, the results may be biased, thus affecting outcomes through causal pathways other than exposure and violating the instrumental variable assumptions [21]. Therefore, we compared IVW results with other established MR methods whose estimates are known to be relatively robust to horizontal pleiotropy, albeit at the expense of reduced statistical power [22]. MR Egger regression based on the assumption of instrument strength independent of direct effect (InSIDE), allowed free estimation of the intercept as an indicator of mean pleiotropic deviation [21]. If the intercept item is zero, it means that horizontal pleiotropy does not exist, and the MR‒Egger regression result is consistent with IVW [21]. The weighted median method selects the median MR estimate as the causal estimate [23]. Weighted model estimates were found to have greater power to detect causal effects, less bias, and a lower Type I error rate than MR‒Egger regression if the InSIDE assumption was violated [24]. We also used MR-PRESSO (Pleiotropic Residuals and Outliers) [25] to detect and remove any significant outliers that reflected possible pleiotropic bias in all reported results. However, this outlier test requires at least 50% of the gene variants to be a valid tool and relies on the InSIDE assumption [25].

Cochran’s IVW Q statistic was used to quantify IV heterogeneity. Furthermore, to identify potentially heterogeneous SNPs, a “leave-one-out” analysis was performed by sequentially omitting each instrumental SNP. To assess the causal relationship between the gut microbiota and cardiovascular disease, we also performed reverse MR analysis of bacteria that were found to be causally associated with cardiovascular disease in the forward MR analysis. The methodology and settings used were consistent with forward MR.

All statistical analyses were performed using R version 4.2.1 and the “TwoSampleMR” package. The package harmonizes exposure and outcome datasets and contains information on SNPs, alleles, effect sizes (odds ratios [OR] converted to β statistics by log transformation), standard errors, *P* values, selected exposure instruments and information on effect allele frequencies.

## Results

According to the selection criteria of IVs, a total of 522 SNPs used as IVs for 121 bacterial genera had a causal relationship between gut microbiota and cardiovascular diseases. Details about the selected instrumental variables are shown in Additional file (Table S1).

### Gut microbiota and atrial fibrillation

#### Causal effect of gut microbiota on atrial fibrillation

**Fig. 2:**
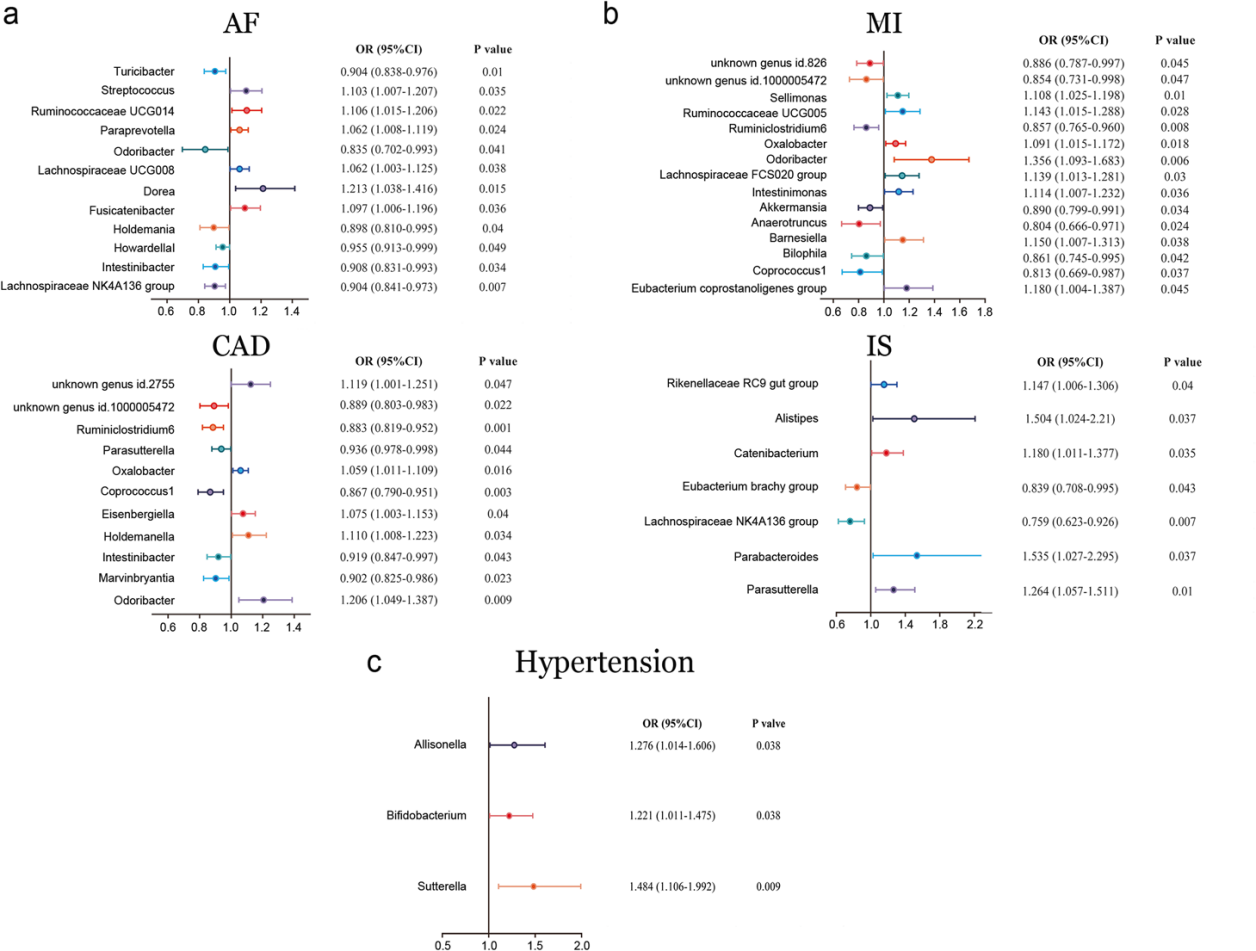
The magnitude of the effect of each gut microbiota on cardiovascular diseases. A protective causal effect is present when the OR is < 1, whereas a causal pathogenic effect is present when the OR is > 1. **a** The effect of each gut microbiota on atrial fibrillation and coronary artery disease. **b** The effect of each gut microbiota on myocardial infarction, and ischemic stroke. **c** The effect of each gut microbiota on hypertension

**Fig. 3:**
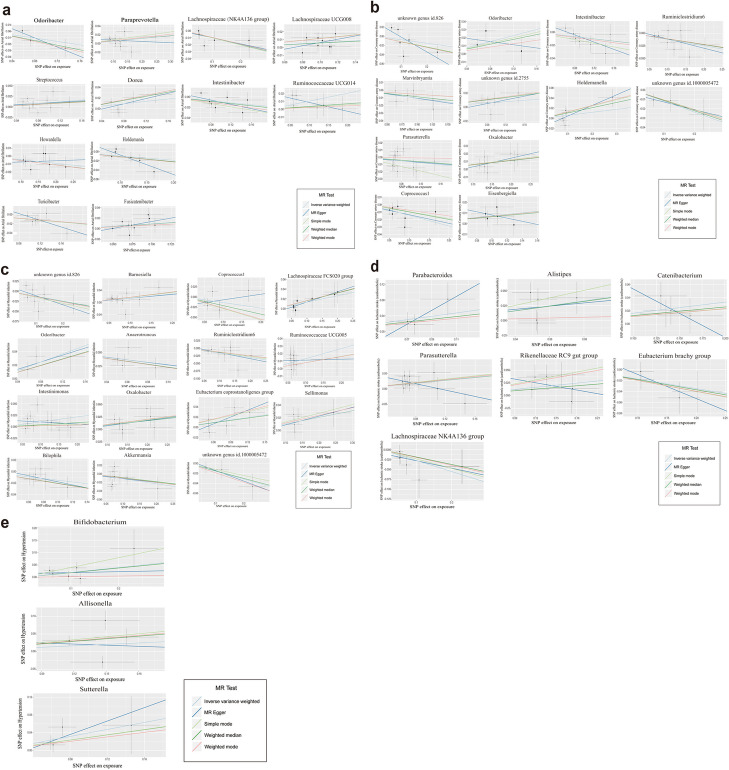
Scatter plots of each gut microbiota associated with cardiovascular diseases. **a** Atrial fibrillation. **b** Coronary artery disease. **c** Myocardial infarction. **d** Ischemic stroke. **e** Hypertension

As shown in Table S1, Figs. 2a and 3a, 12 bacterial genera, including *Dorea*, *Fusicatenibacter*, *Holdemania*, *Howardella*, *Intestinibacter*, *Lachnospiraceae NK4A136 group*, *Lachnospiraceae UCG008*, *Odoribacter*, *Paraprevotella*, *Ruminococcaceae UCG014*, *Streptococcus*, and *Turicibacter* were found to be associated with atrial fibrillation in at least one MR method.

The IVW estimate indicated that *Howardella* had a protective effect against atrial fibrillation (OR = 0.955, 95% CI: 0.913–0.999, *P* = 0.05), and *Intestinibacter* (OR = 0.908, 95% CI:0.831–0.993, *P* = 0.03), *Lachnospiraceae* (NK4A136 group) (OR = 0.904, 95% CI:0.841–0.973, *P* = 0.007), and *Turicibacter* (OR = 0.904, 95% CI: 0.838–0.976, *P* = 0.01) also had protective effects against AF. On the other hand, *Fusicatenibacter* (OR = 1.097, 95% CI: 1.006–1.196, *P* = 0.04), *Lachnospiraceae* UCG008 (OR = 1.062, 95% CI: 1.003–1.125, *P* = 0.04), *Paraprevotella* (OR = 1.062, 95% CI: 1.008–1.119, *P* = 0.02), *Ruminococcaceae* UCG014 (OR = 1.106, 95% CI: 1.015–1.206, *P* = 0.02), and of *Streptococcus* (OR = 1.103, 95% CI: 1.007–1.207, *P* = 0.03) had anti-protective effects on AF. Although IVW estimates did not support a causal relationship between AF and *Dorea*, *Holdemania* or *Odoribacter*, weighted median approach estimates suggested that *Dorea* (OR, 1.213; 95% CI: 1.038–1.416, *P* = 0.01), *Holdemania* (OR, 0.898; 95% CI: 0.810–0.995, *P* = 0.04) and *Odoribacter* (OR, 0.835; 95% CI: 0.710–0.993, *P* = 0.04) were causally associated with AF.

#### Evaluation of assumptions and pleiotropy heterogeneity analyses

**Fig. 4:**
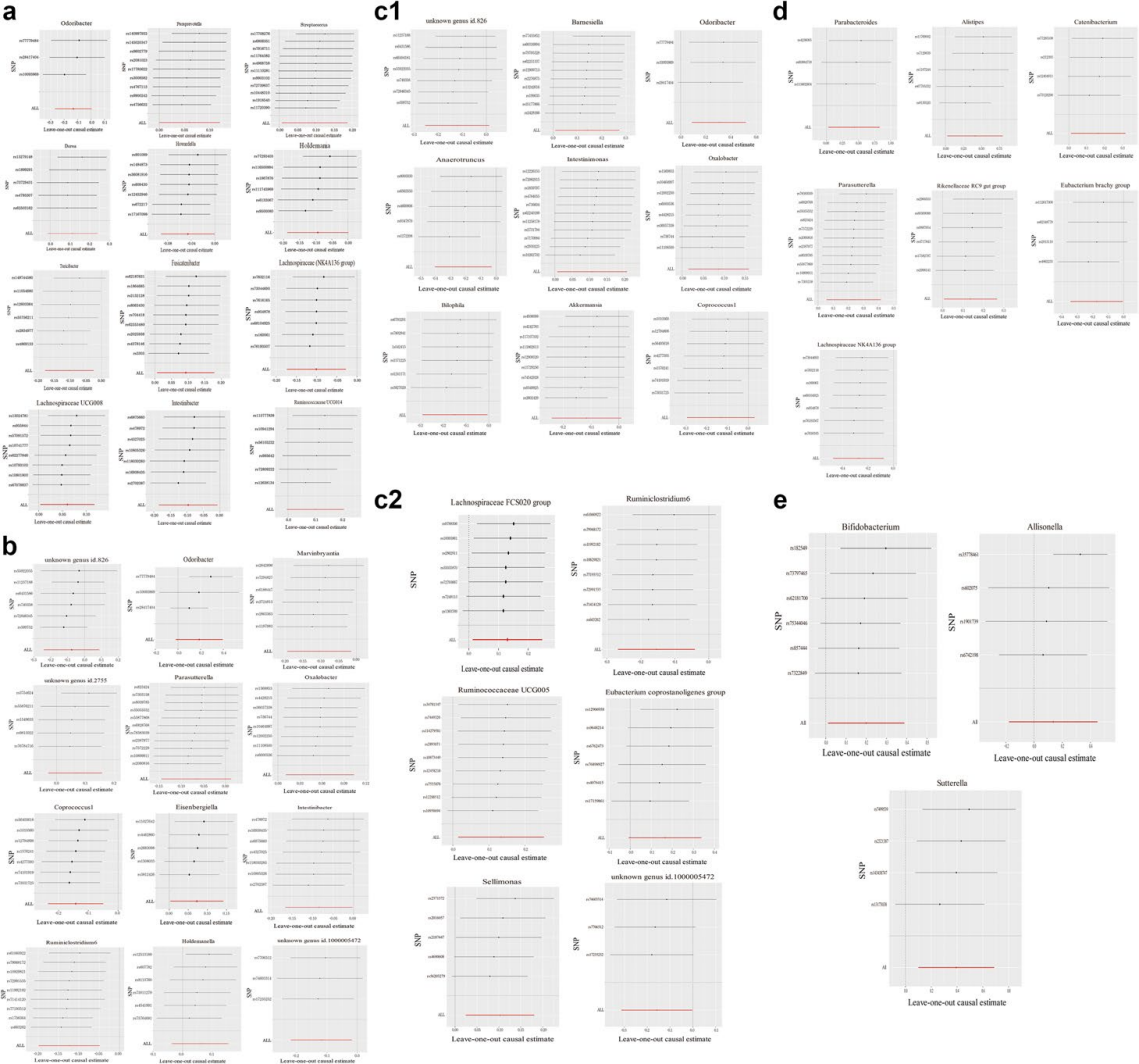
Leave-one-out plot to visualize the causal effect of each gut microbiota associated with cardiovascular diseases when one SNP is omitted. **a** Atrial fibrillation. **b** Coronary artery disease. **c1**-**2** Myocardial infarction. **d** Ischemic stroke. **e** Hypertension

No horizontal pleiotropy was observed in the intercept of the MR Egger regression (*Dorea*, *P* = 0.62; *Fusicatenibacter*, *P* = 0.43; *Holdemania*, *P* = 0.12; *Howardella*, *P* = 0.54; *Intestinibacter*, *P* = 0.5; *Lachnospiraceae* NK4A136 group, *P* = 0.82; *Lachnospiraceae* UCG008, *P* = 0.55; *Odoribacter*, *P* = 0.46; *Paraprevotella*, *P* = 0.4; *Ruminococcaceae* UCG014, *P* = 0.09; *Streptococcus*, *P* = 0.88; *Turicibacter*, *P* = 0.28), indicating that the causal effects were not affected by pleiotropy. In addition, no heterogeneity was observed in our study (*Dorea*, *P* = 0.28, *I*^2^ = 21.8%; *Fusicatenibacter*, *P* = 0.42, *I*^2^ = 1.3%; *Holdemania*, *P* = 0.16,* I*^2^ = 37.4%; *Howardella*, *P* = 0.56, *I*^2^ = 0; *Intestinibacter*, *P* = 0.27,* I*^2^ = 21.3%; *Lachnospiraceae* NK4A136 group, *P* = 0.76,* I*^2^ = 0; *Lachnospiraceae* UCG008, *P* = 0.41,* I*^2^ = 2.5%; *Odoribacter*, *P* = 0.36,* I*^2^ = 3.1%; *Paraprevotella*, *P* = 0.21,* I*^2^ = 26%; *Ruminococcaceae* UCG014, *P* = 0.19,* I*^2^ = 33.5%; *Streptococcus*, *P* = 0.12,* I*^2^ = 35%; *Turicibacter*, *P* = 0.37,* I*^2^ = 6.5%; Table 1). After removing each SNP one at a time, the remaining SNPs were again systematically subjected to MR analysis (Fig. 4a). The results remained consistent, indicating a significant causal relationship between the calculated results for all SNPs. Furthermore, no dominant SNPs were observed in the gut microbiota and the previous MR results were considered to be valid.

**Table 1:** Effect of cardiovascular diseases on gut microbiota

Exposure	Outcome	Methods	Number of SNPs	OR (95% CI)	Beta (SE)	*P*-value	Pleiotropy	Heterogeneity	
							*P*-value	*I*^*2*^ (%)	*P*-value
AF	Dorea	MR Egger	96	1.067 (1.001–1.137)	0.06 (0.03)	0.05	0.09	0	0.82
AF	Streptococcus	IVW	96	0.964 (0.931–0.998)	-0.04 (0.02)	0.04	0.3	2.9	0.4
IS	Alistipes	IVW	4	0.930 (0.869–0.995)	-0.07 (0.03)	0.04	0.79	0	0.64
IS	Eubacterium brachy group	IVW	4	0.846 (0.733–0.977)	-0.17 (0.07)	0.02	0.14	52.4	0.1
IS	Rikenellaceae RC9 gut group	IVW	4	0.855 (0.731–0.999)	-0.16 (0.08)	0.05	0.37	0	0.48

### Gut microbiota and coronary artery disease

#### Causal effect of gut microbiota on coronary artery disease

The results showed that *Coprococcus1* (OR, 0.867; 95% CI:0.790–0.952, *P* = 0.003, Fig. 2a), *Intestinibacter* (OR, 0.919; 95% CI:0.847–0.997, *P* = 0.04), *Marvinbryantia* (OR, 0.901; 95% CI;0.825–0.986, *P* = 0.02), *Parasutterella* (OR, 0.936; 95% CI:0.878–0.998, *P* = 0.04), *Ruminiclostridium6* (OR, 0.883; 95% CI:0.819–0.952, *P* = 0.001) and unknown genus id.1000005472 (OR, 0.889; 95% CI:0.803–0.983, *P* = 0.02) were protective factors against CAD based on IVW. Using the same method, we found that *Eisenbergiella* (OR, 1.075; 95%CI;1.003–1.153, *P* = 0.04), *Odoribacter* (OR, 1.206; 95% CI:1.049–1.387, *P* = 0.009), and *Oxalobacter* (OR, 1.059; 95%CI:1.011–1.109, *P* = 0.02) were risk factors for CAD. In addition to the IVW method, the weighted median method revealed that *Holdemanella* (OR, 1.110; 95% CI:1.009–1.222, *P* = 0.03) and the unknown genus id.2755 (OR, 1.119; 95% CI:1.001–1.251, *P* = 0.05) had deleterious effects on CAD. A scatterplot of each gut microbiota associated with coronary artery disease is shown in Fig. 3b.

#### Evaluation of assumptions and pleiotropy heterogeneity analyses

MR Egger regression revealed no observed horizontal pleiotropy for the intercepts of the microbiota (*Coprococcus1*, *P* = 0.84; *Eisenbergiella*, *P* = 0.53; *Holdemanella*, *P* = 0.24; *Intestinibacter*, *P* = 0.23; Marvinbryantia, *P* = 0.93; *Odoribacter*, *P* = 0.56; *Oxalobacter*, *P* = 0.63; *Parasutterella*, *P* = 0.94; *Ruminiclostridium6*, *P* = 0.58; unknown genus id.1000005472, *P* = 0.77; unknown genus id.2755, *P* = 0.76), thus indicating that the causal effects were not affected by pleiotropy. Furthermore, no significant heterogeneity was observed in the effects of the microbiota on CAD (*Coprococcus1*, *P* = 0.6, *I*^2^ = 0; *Eisenbergiella*, *P* = 0.72, *I*^2^ = 0; *Holdemanella*, *P* = 0.07, *I*^2^ = 51%; *Intestinibacter*, *P* = 0.46,* I*^2^ = 0; *Marvinbryantia*, *P* = 0.65, *I*^2^ = 0; *Odoribacter*, *P* = 0.11, *I*^2^ = 54.7%; *Oxalobacter*, *P* = 0.84, *I*^2^ = 0; *Parasutterella*, *P* = 0.13, *I*^2^ = 33.6%; *Ruminiclostridium6*, *P* = 0.33, *I*^2^ = 12%; unknown genus id.1000005472, *P* = 0.87, *I*^2^ = 0; unknown genus id.2755, *P* = 0.3, *I*^2^ = 17.6%; Table 1). Leave-one-out analysis revealed that the results were robust (Fig. 4b).

### Gut microbiota and myocardial infarction

#### Causal effect of gut microbiota on myocardial infarction

Our study suggested that *Akkermansia* (OR, 0.890; 95% CI:0.799–0.991, *P* = 0.03; Fig. 2b) *Anaerotruncus* (OR, 0.804; 95% CI:0.666–0.971, *P* = 0.02), *Bilophila* (OR, 0.861; 95% CI:0.745–0.995, *P* = 0.04), *Ruminiclostridium6* (OR, 0.857; 95% CI:0.765–0.960, *P* = 0.008), unknown genus id.1000005472 (OR, 0.854; 95% CI:0.731–0.998, *P* = 0.05), and unknown genus id.826 (OR, 0.886; 95% CI:0.787–0.997, *P* = 0.05) had a protective effect against myocardial infarction according to the IVW method. We also found that several genera had were risk factors for MI, including *Barnesiella* (OR, 1.150; 95% CI:1.007–1.313, *P* = 0.04), *Eubacterium coprostanoligenes* group (OR, 1.18; 95% CI:1.004–1.387, *P* = 0.05), *Intestinimonas* (OR, 1.114; 95% CI:1.007–1.232, *P* = 0.04), *Lachnospiraceae* FCS020 group (OR,1.139; 95% CI:1.013–1.281, *P* = 0.03), *Odoribacter* (OR,1.356; 95% CI:1.093–1.683, *P* = 0.006), *Oxalobacter* (OR,1.091; 95% CI:1.015–1.172, *P* = 0.02), *Ruminococcaceae* UCG005 (OR,1.143; 95% CI:1.015–1.288, *P* = 0.03) and *Sellimonas* (OR,1.108; 95% CI:1.025–1.198, *P* = 0.01). In addition to the IVW method, *Coprococcus1* (OR, 0.813; 95% CI: 0.669–0.987, *P* = 0.04) was found to have a positive effect on MI according to the weighted median method. A scatterplot of each gut microbiota associated with myocardial infarction is shown in Fig. 3c.

#### Evaluation of assumptions and pleiotropy heterogeneity analyses

MR Egger regression revealed no observed horizontal pleiotropy for the intercepts of the microbiota (Akkermansia, *P* = 0.91; Anaerotruncus, *P* = 0.98; Barnesiella, *P* = 0.96; Bilophila, *P* = 0.78; Coprococcus1, *P* = 0.3; Eubacterium coprostanoligenes group, *P* = 0.35; Intestinimonas, *P* = 0.28; Lachnospiraceae FCS020 group, *P* = 0.51; Odoribacter, *P* = 0.91; Oxalobacter, *P* = 0.9; Ruminiclostridium6, *P* = 0.74; Ruminococcaceae UCG005, *P* = 0.43; Sellimonas, *P* = 0.88; unknown genus id.1000005472, *P* = 0.78; and unknown genus id.826, *P* = 0.52), indicating that the causal effects were not affected by pleiotropy. Furthermore, no significant heterogeneity was observed in the relationships between the microbiota and MI (Akkermansia, *P* = 0.21, *I*^2^ = 26.8%; Anaerotruncus, *P* = 0.37, *I*^2^ = 5.6%; Barnesiella, *P* = 0.2, *P* = 26.7%; Bilophila, *P* = 0.84, *I*^2^ = 0; Coprococcus1, *P* = 0.25, *I*^2^ = 23.7%; Eubacterium coprostanoligenes group, *P* = 0.33, *I*^2^ = 12.9%; Intestinimonas, *P* = 0.36,* I*^2^ = 8.6%; Lachnospiraceae FCS020 group, *P* = 0.94, *I*^2^ = 0; Odoribacter, *P* = 0.66, *I*^2^ = 0%; Oxalobacter, *P* = 0.67,* I*^2^ = 0; Ruminiclostridium6, *P* = 0.57,* I*^2^ = 0; Ruminococcaceae UCG005, *P* = 0.95,* I*^2^ = 0; Sellimonas, *P* = 0.43,* I*^2^ = 0; unknown genus id.1000005472, *P* = 0.85,* I*^2^ = 0; and unknown genus id.826, *P* = 0.26, *I*^2^ = 21.6%; Table 1). Leave-one-out analysis showed revealed that the results were robust (Fig. 4c1-2).

### Gut microbiota and ischemic stroke (cardioembolic)

#### Causal effect of gut microbiota on ischemic stroke (cardioembolic)

Our study showed that the *Eubacterium brachy* group (OR, 0.839; 95% CI:0.708–0.995, *P* = 0.04; Fig. 2b) and *Lachnospiraceae* (NK4A136 group) (OR, 0.759; 95% CI:0.623–0.926, *P* = 0.007) had a protective effect on ischemic stroke (cardiogenic embolism). *Alistipes* (OR, 1.504; 95% CI:1.024–2.210, *P* = 0.04), *Catenibacterium* (OR, 1.180; 95% CI:1.011–1.377, *P* = 0.04), *Parabacteroides* (OR, 1.535; 95% CI:1.027–2.295, *P* = 0.04), *Parasutterella* (OR, 1.264; 95% CI:1.057–1.512, *P* = 0.01), and *Rikenellaceae* RC9 gut group (OR, 1.147; 95% CI:1.006–1.306, *P* = 0.04) had harmful effects on ischemic stroke.

A scatterplot of each gut microbiota associated with ischemic stroke is shown in Fig. 3d.

#### Evaluation of assumptions and pleiotropy heterogeneity analyses

MR Egger regression revealed no observed horizontal pleiotropy for the intercepts of the microbiota (*Alistipes*, *P* = 0.94; *Catenibacterium*, *P* = 0.48; *Eubacterium brachy* group, *P* = 0.5; *Lachnospiraceae* NK4A136 group, *P* = 0.57; *Parabacteroides*, *P* = 0.52; *Parasutterella*, *P* = 0.06; *Rikenellaceae* RC9 gut group, *P* = 0.39), indicating that the causal effects were not affected by pleiotropy. Furthermore, no significant heterogeneity was observed in the relationships between the microbiota and ischemic stroke (*Alistipes*, *P* = 0.16, *I*^2^ = 39%; *Catenibacterium*, *P* = 0.7, *I*^2^ = 0; *Eubacterium brachy* group, *P* = 0.73,* I*^2^ = 0; *Lachnospiraceae* NK4A136 group, *P* = 0.85, *I*^2^ = 0; *Parabacteroides*, *P* = 0.56, *I*^2^ = 0; *Parasutterella*, *P* = 0.31, *I*^2^ = 13.6%; *Rikenellaceae* RC9 gut group, *P* = 0.51, *I*^2^ = 0). Leave-one-out analysis revealed that the results were robust (Fig. 4d).

### Gut microbiota and hypertension

#### Causal effect of gut microbiota on hypertension

The IVW method revealed that *Bifidobacterium* (OR, 1.221; 95% CI:1.011–1.475, *P* = 0.04; Fig. 2c), and *Sutterella* (OR, 1.484; 95% CI:1.106–1.992, *P* = 0.009) had negative causal effects on hypertension; furthermore, the weighted median method revealed that *Allisonella* (OR, 1.276; 95% CI: 1.014–1.606, *P* = 0.04) had a negative causal effect on hypertension. A scatterplot of each gut microbiota associated with hypertension is shown in Fig. 3e.

#### Evaluation of assumptions and pleiotropy heterogeneity analyses

MR Egger regression revealed no observed horizontal pleiotropy for the intercepts of the microbiota (*Allisonella*, *P* = 0.88; *Bifidobacterium*, *P* = 0.58; *Sutterella*, *P* = 0.67), indicating that the causal effects were not affected by pleiotropy. Additionally, no significant heterogeneity was observed in the relationships between the microbiota and hypertension (*Bifidobacterium*, *P* = 0.46, *I*^2^ = 0; *Sutterella*, *P* = 0.56, *I*^2^ = 0) except *Allisonella* (*P* = 0.001, *I*^2^ = 81%). Leave-one-out analysis s revealed that the results were robust (Fig. 4e).

### Causal effect of cardiovascular diseases on gut microbiota

Inverse MR analysis revealed that some cardiovascular diseases had causal effects on gut microbiota. We found that AF had an adverse effect on *Streptococcus* (OR, 0.964; 95% CI:0.931–0.998, *P* = 0.04), and we observed a similar trend for *Dorea* (OR, 1.067; 95% CI:1.001–1.137, *P* = 0.5). We also found that ischemic stroke had a negative effect on *Alistipes* (OR, 0.930; 95% CI:0.869–0.995, *P* = 0.04), *Eubacterium brachy* group (OR, 0.846; 95% CI:0.733–0.977, *P* = 0.02), and *Rikenellaceae* RC9 gut group (OR, 0.855; 95% CI:0.731–0.999, *P* = 0.05). MR Egger regression revealed no observed horizontal pleiotropy for the intercepts of the CVDs (all *P* > 0.05), and no significant heterogeneity were observed (all *P* > 0.05; Table 1).

### KEGG and Go-enrichment pathway analysis

The enrichments of biological processes (BPs), cellular components (CCs), and molecular function (MFs) based on gene ontology analysis showed that the gut microbiota mainly effected on the insulin secretion, circadian entrainment and aldosterone synthesis and secretion, which potentially suggest the way that target genes affect metabolism (Fig. S1a and b). In detail, BP analysis shows a strong association between target genes and the tissue homeostasis and integrin-mediated signaling pathways, which may involve protein kinase binding, integrin binding and so forth from MF analysis.

## Discussion

CVD is a common, age- and lifestyle-related disease with significant morbidity and mortality. Therefore, early identification, and treatment of risk factors for CVD can reduce the incidence of the disease in the population, thereby improving population health. Although the gut microbiota has been shown to participate in many cellular mechanisms, such as immune regulation, apoptosis, and ferroptosis [26–28], clinical studies [29] have shown correlations between the gut microbiota and disease rather than causal relationship. Romano et al. [30] point that phenylacetylgutamine (PAGln), a gut microbiota metabolite, associated with the presence of heart failure, could be a potential therapeutic target to modulate heart failure; however, this study did not connect metabolites to certain gut microbiota, it is therefore necessary to find out which gut microbiota play a major role in PAGln production in order to carry out precise intervention. Guo et al. [31] shows that intermittent fasting can alter gut microbial community composition and related metabolic pathways, which is associated with mitigating cardiometabolic risk factors. This means that dietary habits are important factors in maintaining cardiac health, and different diets can alter the abundance of gut microbiota and the concentration of its associated metabolites. Neither study mentions specific microbiota associated with disease, but in our study, we present a list of gut microbiotas that contribute to CVD. If the relationship between gut microbiota and CVD is not causal, recommendations for maintaining diversity and stability in gut microbiota for the prevention and treatment of CVD are limited. To strengthen causal inferences, we employ a genetic information approach. Using MR in combination with genetic instruments selected from large-scale GWAS, we found evidence supporting protective and anti-protective relationships between the gut microbiota and CVD.

Our results extend the current literature. To our knowledge, this is the first article to identify causal associations between gut microbiota genera and cardiovascular disease. Previous studies were conducted based on diseased populations, and they only showed that people suffering from a certain disease had different proportions of relevant flora. However, the current study used SNPs as IVs, thus eliminating the interference of confounding factors, instruments, equipment, operations, sampling, and other potential factors influencing flora detection.

Based on our MR study, an unbiased examination of the causal influence of the gut microbiota on cardiovascular disease was performed. Six bacterial genera had protective effects against atrial fibrillation, including *Howardella*, *Intestinibacter*, *Lachnospiraceae* (NK4A136 group), *Turicibacter*, *Holdemania* and *Odoribacter*, suggesting that these genera are probiotics. Another 6 bacterial genera showed pathogenic effects on AF, including *Fusicatenibacter*, *Lachnospiraceae* UCG008, *Paraprevotella*, *Ruminococcaceae* UCG014, *Streptococcus*, and *Dorea*. Six bacterial genera showed protective effects against coronary artery disease, including *Coprococcus1*, *Intestinibacter*, *Marvinbryantia*, *Parasutterella*, *Ruminiclostridium6* and unknown genus id.1000005472. Five bacterial genera were observed to have a negative effect on CAD, including *Eisenbergiella*, *Odoribacter*, *Oxalobacter*, *Holdemanella*, and the unknown genus id.2755. Seven bacterial genera were shown to have a protective effect against myocardial infarction, including *Akkermansia*, *Anaerotruncus*, *Bilophila*, *Ruminiclostridium6*, unknown genus id.1000005472, unknown genus id.826, and *Coprococcus1*. Eight bacterial genera were found to have negative effects on MI, including *Barnesiella*, *Eubacterium coprostanoligenes group*, *Intestinimonas*, *Lachnospiraceae* FCS020 group, *Odoribacter*, *Oxalobacter*, *Ruminococcaceae* UCG005, and *Sellimonas*. Two bacterial genera were found to have protective effects against ischemic stroke (cardiogenic embolism), including *Eubacterium brachy group* and *Lachnospiraceae* (NK4A136 group). In addition, five bacterial genera were associated with IS progression, including *Alistipes*, *Catenibacterium*, *Parabacteroides*, *Parasutterella* and *Rikenellaceae* (RC9 gut group), suggesting that these are harmful bacteria. We also found that 3 bacterial genera, including *Bifidobacterium*, *Sutterella*, and *Allisonella*, were associated with the development of hypertension. This is the first MR study to combine gut microbiota with genomics to assess the causal impact of gut microbiota on CVDs and some of these risk factors. Our study provides new insights into the role of gene‒environment interactions in the development and progression of human diseases.

At present, the influence of gut microbiota on AF remains to be further studied. Changes in Dorea have now been found to be associated with Takayasu arteritis [32]. However, this conclusion can only illustrate the association, because these patients with Takayasu arteritis may also face AF. Another study found that *Dorea* abundance was altered in hypertensive patients with AF [33], but we did not find a causal relationship between *Dorea* and hypertension. This may be because changes in *Dorea* lead to changes in gut metabolites, and an imbalance between these metabolites, such as saturated and unsaturated fatty acids, can lead to interactions between metabolites or with other flora, leading to other diseases, but *Dorea* has no direct causative relationship with these diseases. In our study, we found that the relationship between *Dorea* and AF is reciprocal, so it is expected to be a gut microbiota target for the treatment of AF. Although there is a bidirectional interaction between AF and *Streptococcus*, the abundance of this group decreases as AF progresses, thus inhibiting the pathogenic role of *Streptococcus*. Microorganisms such as *Lachnospiraceae* were found to be causative in AF, associated with a reduction in short-chain acids and anti-inflammatory components [34]. In our study, 6 gut microbiotas were found to be causally associated with AF for the first time: *Fusicatenibacter*, *Holdemania*, *Howardella*, *Intestinibacter*, *Ruminococcaceae* UCG014 and *Turicibacter*. Furthermore, most of them had protective effects on AF, with the exception of *Fusicatenibacter* and *Ruminococcaceae* UCG014. In the elderly population, AF is a common disease, that not only causes heart failure, but also increases the risk of stroke; therefore, if we can accurately predict AF, we can implement timely interventions to reduce the risk of complications and the emergence of symptoms. However, extensive research is still needed before microbiome sequencing can be used for the prevention and targeted treatment of AF, such as whether there is crosstalk among the metabolites produced between the various bacterial groups.

For coronary artery disease and its acute myocardial infarction, an interesting phenomenon was found in our study; that is, the intestinal pathogenic flora of coronary artery disease or myocardial infarction did not have the same flora except for *Odoribacter* and *Oxalobacter*. It has been reported that when the abundance of *Odoribacter* is increased in the intestinal tract of inflammatory bowel disease, this microbe can maintain intestinal homeostasis by, for example, reducing the level of reactive oxygen species [35]. However, the abundance of *Odoribacter* was positively correlated with CAD and MI progression, so the mechanism of *Odoribacter* involvement awaits further investigation, and it may become a therapeutic target against CAD and the consequent AMI. In addition, we found that many gut floras belonged to the probiotics of both CAD and MI, including some unknown genera, *Ruminiclostridium 6*, and *coprococcus1*, but some others were different. *Ruminiclostridium 6* has been reported to be protective against systemic lupus erythematosus, possibly because it can participate in anti-inflammatory processes [36]. This could strengthen the conclusion that the progression of coronary heart disease and myocardial infarction is also influenced by inflammatory factors. However, pathogenic bacteria for MI are different from those for CAD, which means that the alteration of the metabolic state in AMI is totally different. We believe that changes in gut metabolic status in CAD are chronic, but gut flora in AMI may contribute to AMI progression due to rapid changes in gut metabolite levels. This may be why CAD and MI do not share all pathogenic bacteria. Moderate to intense physical activity can improve heart function and increase the abundance of gut microbes such as *Akkermansia*, a study reported [37], which means that daily exercise can also alter the gut microbiota, so we can reduce AMI risk through regular physical activity. Furthermore, when there is a change in daily diet, the gut microbiome, such as *Bilophila*, can quickly respond to this change [38]. Therefore, a healthy diet is recommended if we want to reduce the risks of AMI or CAD.

Ischemic stroke (cardiogenic embolism) is the leading cause of disability and the second leading cause of death worldwide, especially in people older than 65 [39]. In our study, 5 pathogenic bacteria were found to be associated with the progression of cardiogenic stroke. We found a bidirectional causal relationship between *Alistipes* and IS, with increased *Alistipes* abundance leading to IS and IS reducing *Alistipes* abundance. This is not consistent with the result of a previous study: IS can induce intestinal microbial disturbance by enriching microorganisms such as *Alistipes* [40]. We believe that this phenomenon may be due to the combined effect of other intestinal pathogens and opportunistic microorganisms, and the changes in the abundance of these flora not only destabilize the intestinal microenvironment but also lead to the expression of certain bacterial metabolic profiles and the proliferation of abnormal flora. Gut microbiota, such as the *Rikenellaceae* RC9 gut group, are involved in Parkinson’s disease (PD) by altering gut branched chain and aromatic amino acid ratios to modulate immune responses [41]. Increased abundance of the *Rikenellaceae* RC9 gut group was associated with PD pathogenesis [41], similar to our finding that this group is the causative agent in IS. In addition, we found that one of IS’s protective gut microbiota (*Eubacterium brachy* group) has also been reported by other studies to screen for colorectal cancer [42]. Another protective gut microbiota (*Lachnospiraceae* NK4A136 group) for IS has been found to enhance gut barrier function, as supported by a previous study [43]. We found 3 pathogens of hypertension, including 2 newly identified groups (*Bifidobacterium* and *Sutterella*) and *Allisonella*, which is inconsistent with a previous Mendelian randomization study [44] that showed *Allisonella* to be a protective microbiota in hypertension. This may be because we are not using the same measurement method, and the samples come from different sources.

The cohorts used in this study for a genome-wide study of the gut microbiome mainly included Europeans, and most cohorts included either adult or adolescent individuals, suggesting that our findings are evenly represented in European adult or adolescent individuals. Therefore, our findings also apply to adults or adolescents with cardiovascular disease.

Our study also has several limitations. First, although the use of MR to exclude confounding factors improved the accuracy and reliability of the study and yielded interesting results, differences in microbiome structure at multiple taxonomic levels were an important covariate, which was largely driven by heterogeneity between populations and differences in technical solutions. Second, the six GWASs adopted the meta-analytic method by pooling data from several cohorts. This approach works well at capturing ethnic-specific genetic traits but can introduce bias between samples due to overlap. Third, MR analysis often reveals lifetime exposure, so the effect on exposure should be investigated further through randomized controlled trials. Fourth, the survival analysis cannot be measure because of the limited data from the original studies. Finally, participants in these six GWASs were predominantly non-Asian; therefore, our results mainly apply to populations of European and American descent. Hence, these findings should be generalized to other ethnic groups with caution.

We compared our study with other studies; we found that MR could provide a method to connect the gut microbiota and disease without using intestinal metabolites as mediators, which is an innovation compared to previous studies. Some of the protective gut microbiota we identify in this study, such as Marvinbryantia, Parasutterella, and Intestinibacter, will be of great interest if future studies can explore the direct mechanisms of action between microbiota and disease. To note, the gut microbiota is highly complex and requires high-level and in-depth analysis to explore its systemic mechanisms. Traditional microbiome analysis cannot distinguish the specific abundance of each gut microbiota in normal individuals and individuals with CVD. Therefore, it is reasonable to explore machine learning techniques to achieve accurate diagnosis of the gut microbiota of CVD patients. In the future, understanding which genes or metabolites contribute to intestinal metabolic mechanisms may lead to biomarkers of CVD in the gut microbiota; however, it requires highly accurate prediction models. Although the combination of DNN classifiers and VGG network models [45] may give reliable results, it is important to improve the predictive power of ML-based models if we want to develop new strategies for early diagnosis of CVD.

## Conclusion

This study demonstrated protective and pathogenic relationships between gut microbiota and cardiovascular diseases; therefore, some genera were identified as promising targets for future treatments. Furthermore, we also found a bidirectional causal relationship between some bacterial genera and atrial fibrillation and ischemic stroke. Overall, this study supports the hypothesis that some gut bacterial genera are involved in CVD progression while others are protective factors against CVDs.

### Supplementary Information

**Additional file 1: Supplementary Fig. S1.** (1a) KEGG and BP analysis. (2a) CC and MF analysis.

**Additional file 2.**

## Data Availability

The original contributions presented in the study are included in the article/Supplementary Material, and further inquiries can be directed to the corresponding authors.
