# Supplementary figures and images for: Assessment of the causal relationship between gut microbiota and cardiovascular diseases: a bidirectional Mendelian randomization analysis
Source: BioData Min. 2024 Feb 26;17:6. doi: 10.1186/s13040-024-00356-2 (PMC10898129; doi:10.1186/s13040-024-00356-2)

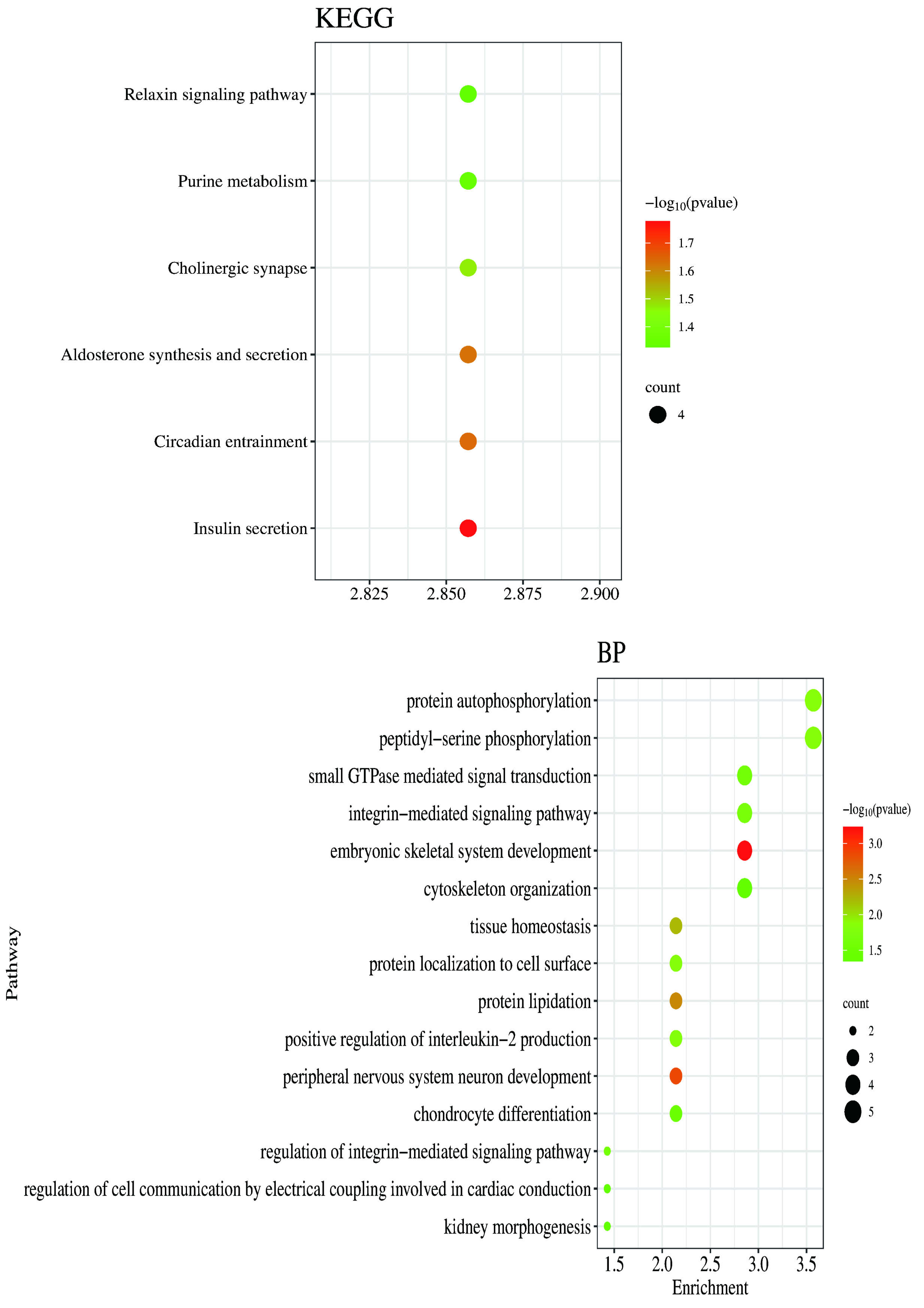

Supplement: Supplementary file 1 — Additional file 1: Supplementary Fig. S1. (1a) KEGG and BP analysis. (2a) CC and MF analysis. [file 13040_2024_356_MOESM1_ESM.zip › FigS1A.tif]

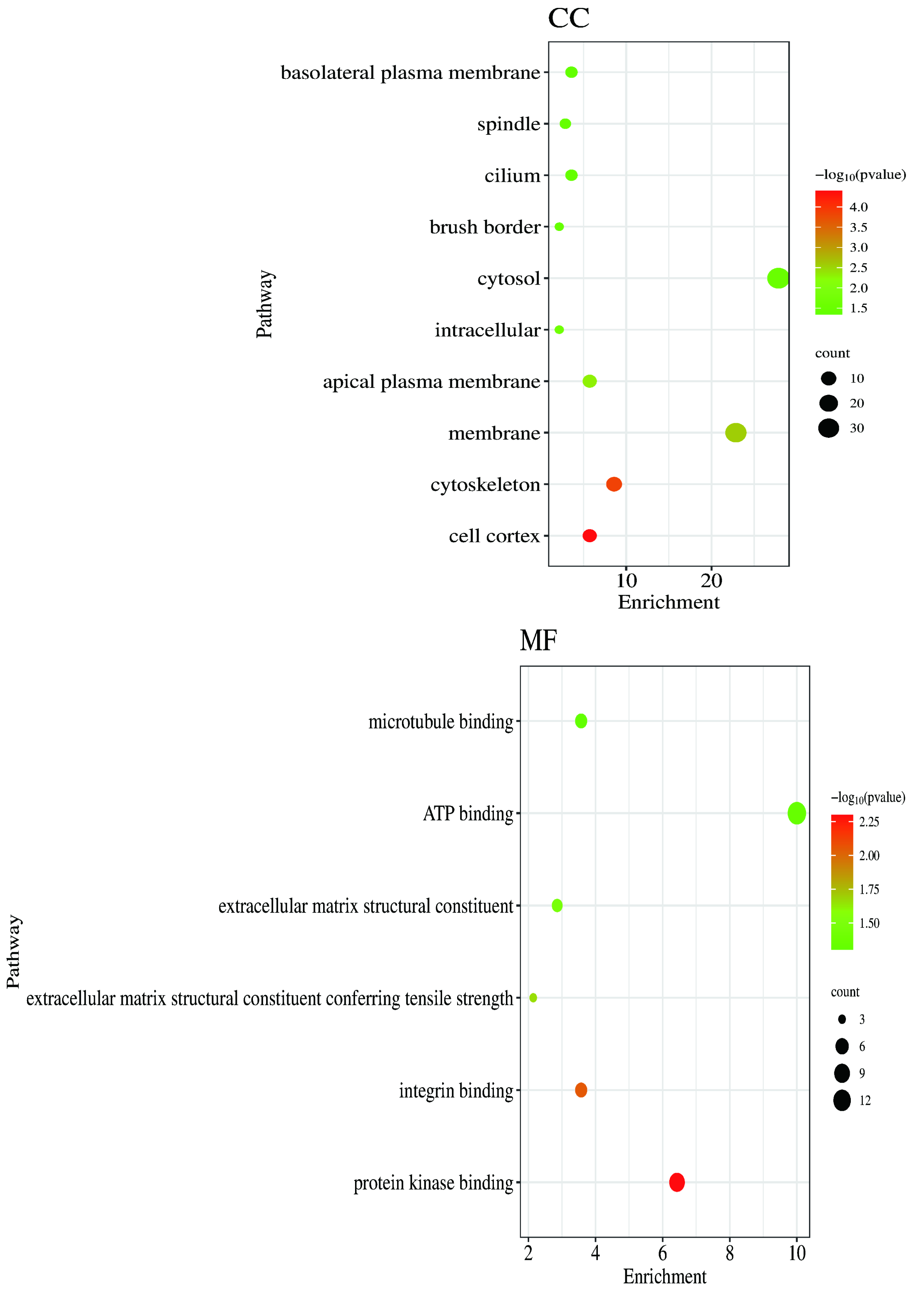

Supplement: Supplementary file 1 — Additional file 1: Supplementary Fig. S1. (1a) KEGG and BP analysis. (2a) CC and MF analysis. [file 13040_2024_356_MOESM1_ESM.zip › FigS1b.tif]
